# Retinopathy of prematurity, a two-year experience at the ROP screening unit from AL-Zahraa Teaching Hospital, AL-Najaf, Iraq

**DOI:** 10.25122/jml-2022-0060

**Published:** 2022-11

**Authors:** Giyathaldeen Thajeel Neamah, Mohammed Qasim Al Nwuaini, Khalid Abbas Abd, Alaa Jumaah Manji Nasrawi, Shamim Riadh Mohammed Hussein

**Affiliations:** 1 Department of Surgery, College of Medicine, Jaber Ibn Hayan University, Najaf, Iraq; 2 Mysan Health Directorate, Maysan, Iraq; 3 Department of Pediatrics, College of Medicine, University of Kufa, Kufa, Iraq; 4 Department of Obstetrics and Gynecology, College of Medicine, Jaber Ibn Hayan University, Najaf, Iraq

**Keywords:** retinopathy of prematurity, ROP, risk factors, premature

## Abstract

This study aimed to assess the incidence of retinopathy of prematurity (ROP) in Al Najaf city, define the risk factors of ROP, and assess the performance of the newly implemented ROP screening. This retrospective study was performed in the ROP screening clinic in Al Najaf city between January 2018 and December 2019. 247 neonates were examined at the ROP screening clinic, with gestational age ≤30 weeks, weight ≤1500 g, other fetal and maternal risk factors, and older or heavier newborns with a complicated course. Out of all the neonates, 90 were enrolled in this study because all others lost contact with the ROP clinic. 62 out of 90 enrolled neonates (69%) were diagnosed with ROP. Of them, 82% had stage 1–2 ROP, and 18% had stage 3–4. We found that the higher gestational age was protective for ROP (P-value=0.012, OR=0.434, CI=0.227–0.829). CPAP carried a higher risk of ROP (P-value=0.072, OR=7.276, CI=0.834–63.441). The P-value was significant for maternal age and premature rupture of membranes (PROM) (P-value=0.028 and 0.01, respectively). This study showed the incidence of ROP in the accepted range compared to other countries with similar resources. Furthermore, there was a strong association between ROP and the following factors: decreased gestational age, CPAP, maternal age, and PROM.

## INTRODUCTION

Retinopathy of prematurity (ROP) is one of the prematurity complications that may lead to blindness. It occurs because of abnormal vascularization of the immature retina. The retina is the inner layer of the eye that receives light and translates it into visual signals analyzed by the brain. In a premature baby, the retinal blood vessels can grow abnormally. Most ROP resolves without residual damage to the retina. However, when it is severe, ROP may pull the retina away or detach it from the wall of the eye and possibly cause blindness [1].

The potential risk factors that may lead to ROP are prematurity, exposure to oxygen in a high percentage and for a long time, low birth weight, septicemia, and congenital heart diseases [2].

The International Classification of Retinopathy of Prematurity was published in 1984, then revised in 2005 (Table 1) [3, 4].

**Table 1:** Classification of retinopathy of prematurity.

**Stage 1**	The demarcation line separates a vascular from a vascularized retina
**Stage 2**	Ridge arising in region of the demarcation line
**Stage 3**	Extra retinal fibrovascular proliferation/neovascularization
**Stage 4**	Partial retinal detachment
**Stage 5**	Total retinal detachment
**Pre-plus disease**	More vascular tortuosity than normal, but insufficient for the diagnosis of plus disease
**Plus disease**	Vascular dilation and tortuosity of at least two quadrants of the eye

**Figure 1:**
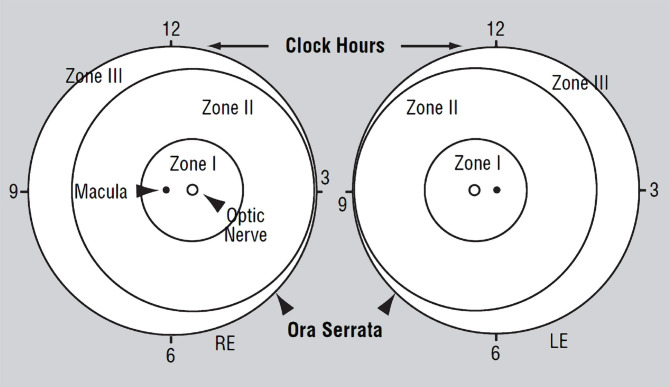
A Diagram of the retina of the right and left eyes [4].

The retina is classified into three zones so that the ophthalmologist can identify the anteroposterior location of ROP at the time of examination, as shown in Figure 1.

Demonstrating zone borders and clock hours are used to describe the location and extent of retinopathy of prematurity [3]. With the improvement of premature neonates and increased survival rate, ROP has become the main cause of childhood blindness and the most potentially preventable. Approximately one and a half million children are blind, and ROP represents about 50,000 cases [5]. This blindness can be avoided by a simple screening test done within a few weeks after birth by an ophthalmologist. On 18 February 1999, the World Health Organization (WHO) adopted a project (VISION 2020), a global initiative that aims to eliminate avoidable blindness by 2020. VISION 2020 targets the control of blindness in children through many steps. In what concerns ROP, the objective of WHO is to ensure that all babies at risk of ROP have a fundus examination by a trained observer 6–7 weeks after birth. Cryo or laser treatment should be provided for all those with threshold disease [6]. Developed countries have adopted strict screening protocols for the early detection and treatment of ROP. However, these standards are still lacking in developing countries despite the higher percentage of preterm labor. The burden of this blindness is set to increase tremendously in these countries if corrective steps are not taken immediately [7]. The differences in prenatal and neonatal care between developed and developing nations can affect the vulnerability of preterm babies concerning ROP and the severity of that disease. Consequently, the inclusion criteria for ROP screening according to United States guidelines might not be fit to diagnose ROP in preterm babies in developing countries, which is why screening recommendations should be designed for the targeted population [8].

The aim of this study was to (1) assess the incidence of ROP in Al Zahraa teaching hospital, Al Najaf, (2) identify risk factors, and (3) assess the performance of the newly implemented ROP screening unit in our hospital (review the criteria for inclusion and discharge from the ROP screening program, human resources, equipment, data basis, the outcome of ROP patients, and treatment options).

## MATERIAL AND METHODS

### Study design

This retrospective cohort study was performed at the ROP Screening Clinic at Al Zahraa Teaching Hospital in Al Najaf Al Ashraf City between January 2018 and December 2019.

### Study population

247 neonates were examined at the ROP screening clinic in the above-specified period. All of them met the following criteria:

- Neonates born at gestational age ≤30 weeks;
- Neonates born with birth weights ≤1500 gm;
- Older or heavier newborns with a complicated medical course, at the discretion of the neonatologist [9] (these criteria were adopted by the ROP screening clinic at Al Zahraa hospital).

Of them, only 90 neonates were enrolled in this study because most candidates lost contact with the ROP clinic or did not agree to participate in the study.

### Ophthalmological examination

The ophthalmological examination was done by preparing the infant with a mydriatic agent combination of tropic amide eye drop 0.4 mg and phenylephrine 0.2 mg three times with intervals of about 10 minutes between each drop. Then the child received a topical anesthetic eye drop, and an examination was done by the fundus camera wide flew 130 degrees (ret-cam shutter) at the ROP screening unit in Al Zahraa teaching hospital by an expert ophthalmologist. Pictures were captured and stored on a computer and interpreted according to the international ROP classification. According to that system, it was organized into staging and managed accordingly and the end of the examination, using a topical antibiotic eye drop (gentamycin eye drop 0.3%) [4].

### Data setting

Neonatal and maternal variables were studied as potential risk factors for the development of ROP, including:

1. Neonatal: gender, birth weight, gestational age, time of O_2_ exposure, length of hospital stays, and type of respiratory support;
2. Maternal: hypertension, diabetes, smoking, anemia, drugs used during pregnancy, infection, assisted conception, mode of delivery, history of premature rupture of the membrane, and chorioamnionitis.

### Statistical analysis

Statistics were done by SPSS statistical software for Windows, V 26. The results are expressed as numbers (N), percentages (%), and means with their respective standard deviation (SDs). Variables with a P value less than 0.05 were considered significant according to logistic regression analyses. The odd ratio (OR) and 95% confidence interval (CI) for each risk factor were determined.

## RESULTS

**Figure 2:**
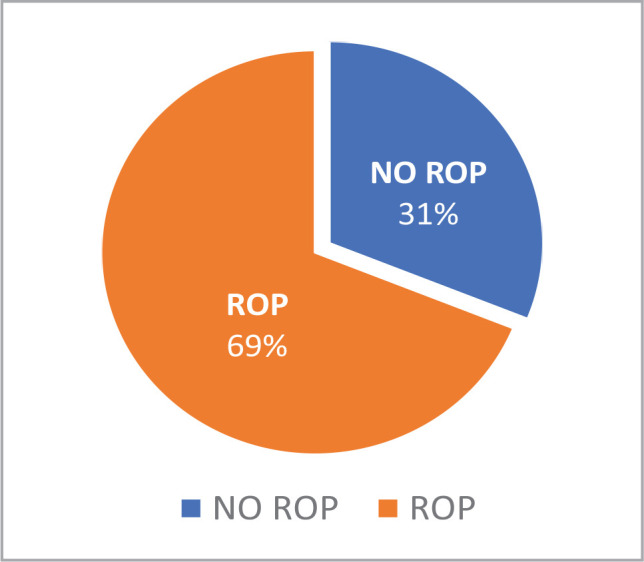
The percentage of ROP-positive neonates.

A total number of 90 neonates were enrolled in the study from January 2018 to December 2019. 62 (69%) of them were diagnosed with ROP by Ret Cam at different stages, as shown in Figure 2.

The patients with ROP were in different stages and zones, as shown in Table 2.

**Table 2:** Stages and zone distribution of ROP-positive patients.

	Stage 1–2	Stage 3–5	Total
	N (%)	N (%)	N (%)
Zone 1	10 (16)	7 (11)	17 (27)
Zone 2	31(50)	3 (5)	34 (55)
Zone 3	10 (16)	1 (2)	11 (18)
Total	51 (82)	11 (18)	62 (100)

Many fetal and maternal variables were studied as potential causes or risk factors for ROP, as shown in Table 3.

**Table 3:** Potential risk factors for ROP.

Variables				P-value	OR	95% C.I.	
						Lower	Upper
Neonatal	Gender	Male (%)	43 (48)	0.169	0.266	0.040	1.753
		Female (%)	47 (52)				
	GA (weeks) Mean (±)		30.7 (0.7)	0.012	0.434	0.227	0.829
	BW (g) Mean (±)		1500 (35.4)	0.770	1.000	0.998	1.003
	O_2_ exposure time (hours) Mean (±)		254.5 (288.5)	0.658	0.997	0.983	1.011
	Length of hospital stay (days) Mean (±)		14.9 (11.3)	0.222	1.206	0.893	1.631
	Type of respiratory support [N (%)]	Nasal cannula	33 (37%)	0.072	7.276	0.834	63.441
		CPAP	57 (63%)				
		Ventilators	0 (0%)				
Maternal	HTN [N (%)]		11 (12)	0.590	0.406	0.015	10.793
	DM [N (%)]		8 (9)	0.343	9.035	0.096	854.37
	Age (years) Mean (±)		25.6 (2.1)	0.028	1.323	1.031	1.698
	Drugs used [N (%)]		19(21)	0.969	1.071	0.033	34.720
	Smoking [N (%)]		2 (2)	0.999	0.000	0.000	-
	Anemia [N (%)]		65 (72)	0.386	2.511	0.313	20.131
	Infections [N (%)]		49 (54)	0.055	6.686	0.964	46.379
	Assisted conception [N (%)]		9 (10)	0.964	1.090	0.026	46.109
	Mode of Delivery [N (%)]	C/S	54 (60)	0.413	2.945	0.222	39.061
		VD	36 (30)				
	PROM [N (%)]		39 (43)	0.010	0.072	0.010	0.532
	Chorioamnionitis [N (%)]		23 (25.5)	0.535	0.418	0.027	6.559

OR – odd ratio; C.I. – confidence interval.

## DISCUSSION

According to UNICEF, the neonatal mortality rate dropped in the last decade from around 20 to 15/1000 live births in Iraq [10]. However, this significant drop made health authorities face many cases of prematurity complications, ROP being the most tragic one.

From January 2018 until December 2019, 3146 neonates were admitted to the neonatal intensive care unit (NICU) in Al Zahraa Teaching Hospital. ROP was detected in 62 of those eligible for screening criteria (247), so the incidence was 1.97% of the total number (3146). In India, Crystal Le et al. [11] found an incidence of 2.3%. However, his study contained a larger cohort size over more years. The ROP incidence is much lower in the USA, according to Eleonora M Lad et al. [12], which may be attributed to better oxygen therapy control that is not available in our center. We use 100% O_2_ therapy because we have no medical air. Out of the 90 neonates examined for ROP screening, 62 (69%) were diagnosed with varying stages, compared to the study by Bader Al-Qahtani et al. [13] in Saudi Arabia, who found 38.6%, which is much lower than in our study. Furthermore, Bader Al-Qahtani et al. [13] showed that 10.6% of ROP-positive patients had less severe disease, *i.e*., stage three, which is also lower than our finding of 18%. The larger sample size plus the good O_2_ therapy policy used in their NICU may be attributed to this difference. Various neonatal risk factors were studied; of them, we found that the higher gestational age is protective for ROP (P value=0.012, OR=0.434, CI=0.227–0.829). Moreover, continuous positive airway pressure (CPAP) carries a higher risk of ROP (P value=0.072, OR=7.276, CI=0.834–63.441). These findings are similar to a meta-analysis performed in Iran [14]. Body weight showed no significant risk factors, which contradicts the findings of Milad Azami et al. [14]. This may be attributed to the small cohort size and the adopted guidelines, which limit the body weight for enrolled neonates in ROP screening to <1500 g. Therefore, this needs to be revised because a multi-center study by Bas et al. [15] in Turkey showed that 15% of ROP-positive neonates had bodyweights >1500 g. See Table 4 to compare the adopted criteria for inclusion in the ROP screening program in Al Zahraa teaching hospital compared to other centers [16].

**Table 4:** Comparison of ROP screening guidelines adopted in different countries and Al Zahraa Teaching Hospital [16].

Country	BW (g)	GA (weeks)	Other criteria	Initial screening
USA	<1500	<30	Older or heavier infants with unstable clinical course, at the discretion of the neonatologist	<27 weeks GA, screening at 31 weeks PMA; 27–30 weeks GA, screening at 4 weeks chronologic age
UK	<1250	<31	Infants with BW of 1251–1501 or GA of <32 weeks	<27 weeks GA, screening at 30–31 weeks PMA; 27–32 weeks GA, screening at 4–5 weeks chronologic age
Australia	<1250	<31	Unstable course or prolonged oxygen therapy	4 weeks chronologic age, but no earlier than 31 weeks PMA
India	<1750	<34	Screening even for older and heavier infants if high-risk factors present	4 weeks chronologic age; but for GA <28 weeks or weight <1200 g screening at 2–3 weeks after birth
China	<2000	≤34	Any infant, irrespective of BW or GA, if ventilated for >1 week or received supplemental oxygen >30 days	4–6 weeks chronologic age or at 32–34 weeks PMA
South Africa	<1500	<32	1500–2000 g BW with risk factors such as a family history of ROP, cardiac arrest, multiple (>2) blood transfusions, exchange transfusion, or severe HIE	4–6 weeks chronologic age or 31–33 weeks PMA (whichever is later)
Iraq (Al Zahraa teaching hospital)	<1500	<30	Older or heavier infants with unstable clinical course	<27 weeks GA, screening at 31 weeks PMA; 27–30 weeks GA, screening at 4 weeks chronologic age

BW – birth weight; GA – gestational age; HIE – hypoxic-ischemic encephalopathy; PMA – postmenstrual age; ROP – retinopathy of prematurity.

Many fetal risk factors were studied in the Iranian meta-analysis which were not included in our study due to a lack of a detailed database which should be improved to include twin pregnancy, history of blood transfusion, septicemia, phototherapy, APGAR score, IVH, multiple gestations, hypoglycemia. Consequently, after improving our database, these potential fetal risk factors could be studied again with a larger cohort size. Regarding maternal risk factors, the P-value was significant for maternal age and premature rupture of membrane (P-value=0.028 and 0.01, respectively). Bas et al. [15] showed no significance for maternal age; however, other maternal risk factors were significant such as antenatal steroids, preeclampsia, chorioamnionitis, vaginal delivery, and breastfeeding. Premature rupture of membranes (PROM) was protective against ROP (odd ratio 0.072, C.I. 0.010–0.532). This is similar to a study by Lee et al. [17], who investigated pregnancy disorders that may modify the risk for ROP. The standard equipment that should be available at the reference ROP clinic in both the diagnostic department (screening) and therapeutic department includes [18]:

- Diagnostic department: include Ret Cam, drops for pupil dilatation, resuscitation trolley, and sterilization materials;
- Therapeutic department: anti-VEGF material (bevacizumab, Ranibizumab), indirect delivery laser therapy, vitrectomy in late stages.

In what concerns the diagnostic part, after reviewing the ROP clinical standard equipment, our center is fully equipped but with regard to the therapeutic part, we still lack laser therapy and are limited to using only anti-VEGF plus vitrectomy in late stages. The laser has the advantage of less stress on the patient, is easy to administer, has better outcomes, results in less refractive myopic shift, and gives superior visual acuity [19]. In comparison with the criteria in our center to stop ROP follow-up for enrolled candidates, the AAP indications for stopping screening examinations include the following [20]:

- Complete vascularization;
- Zone III vascularization without previous zone I or II ROP;
- PMA of 45 weeks and no pre-threshold disease or worse ROP;
- Regression of ROP.

In our ROP screening clinic, the eye examination is terminated in the following circumstances:

- When there is adequate retinal vascularization to zone III;
- Screening is not stopped before 37 weeks of PMA;
- Infants who show ROP changes at some period during follow-up examination require at least two examinations with signs of regression of ROP.

The paper-based database makes it difficult to trace the patient's condition after treatment and to search for other long-term complications of ROP, whether treated or resolved spontaneously, such as myopia. The center is operated by a well-trained and dedicated ophthalmologist.

One of the limitations of this study was that many neonatal variables which may carry a potential role in the pathogenesis of ROP (such as neonatal sepsis, history of blood transfusion, history of NEC, and neonatal hypoglycemia) were not studied because of a lack of detailed medical record in the NICU. For the same reason, we cannot search for patients with ROP complications who tested negative in our center. Therefore, we cannot comment on the sensitivity of the screening.

### Recommendations

Based on our study, we recommend supplying the NICU with medical air so that 100% O_2_ can be blended with it to decrease the effect of CPAP on the retina, to revise the ROP screening program inclusion criteria to include heavier and older gestational age neonates, to revise the ROP screening program discharge criteria to ensure a more extended period of follow-up, and to implement a digital database containing complete medical records so that we can follow up with the patient accurately.

## CONCLUSIONS

The ROP incidence is in the accepted range compared to countries with similar resources. Smaller gestational age and CPAP therapy carry a higher risk for ROP. Maternal age carries risk factors for ROP, and PROM is a protective factor against ROP.
